# Myocardial T2 mapping using wideband T2 preparation gradient echo readout for patients with implantable cardiac devices at 1.5T

**DOI:** 10.1016/j.jocmr.2026.102717

**Published:** 2026-03-18

**Authors:** Pauline Gut, Hubert Cochet, Thomas Küstner, Guido Caluori, Konstantinos Vlachos, Panagiotis Antiochos, Ambra Masi, Juerg Schwitter, Frederic Sacher, Pierre Jaïs, Matthias Stuber, Aurélien Bustin

**Affiliations:** a Department of Diagnostic and Interventional Radiology, Lausanne University Hospital and University of Lausanne, Lausanne, Switzerland; b IHU LIRYC, Heart Rhythm Disease Institute, UniversitÉ de Bordeaux – INSERM U1045, Avenue du Haut LÉvÊque, Pessac, France; c Faculty of Biology and Medicine, University of Lausanne, UniL, Switzerland; d Department of Cardiovascular Imaging, HÔpital Cardiologique du Haut-LÉvÊque, CHU de Bordeaux, Avenue de Magellan, Pessac, France; e Department of Diagnostic and Interventional Radiology, University Hospital Tuebingen, Tuebingen, Germany; f Cardiovascular Department, Cardiology Division, University Hospital of Lausanne (CHUV), Switzerland; g Director of the CMR Center and the Interventional MRI Center of the University Hospital Lausanne, CHUV, Switzerland; h Department of Cardiac Pacing and Electrophysiology, HÔpital Cardiologique du Haut-LÉvÊque, CHU de Bordeaux, Avenue de Magellan, Pessac, France; i CIBM Center for Biomedical Imaging, Lausanne, Switzerland

**Keywords:** Myocardial T2 mapping, Implantable cardiac devices, Implantable cardioverter defibrillator, Wideband MRI, Denoising

## Abstract

**Background:**

Myocardial T2 mapping enables non-invasive assessment of inflammation and edema. However, in patients with implantable cardiac devices, such as pacemakers or defibrillators (ICDs), off-resonance effects often cause severe image artifacts and inaccurate T2 values.

**Purpose:**

The aim of this study was to develop and evaluate a wideband T2-prepared gradient-echo (GRE) myocardial T2 mapping sequence combined with an advanced patch-based denoising approach, designed to reduce artifacts and improve image quality in device-implanted patients at 1.5T.

**Methods:**

A T2 preparation with wideband adiabatic refocusing pulses (5.0 kHz bandwidth) was integrated into a breath-held 2D GRE T2 mapping sequence (TE = 0/27/55 ms). Patch-based denoising was applied after image reconstruction. The sequence was tested in a phantom, 8 healthy volunteers with and without ICDs placed on their chests, 13 patients without devices, 7 patients with ICDs or pacemakers, and 1 sheep scanned before and after induced myocardial infarction with and without external ICD. The proposed sequence was compared against reference conventional GRE and balanced steady-state free-precession (bSSFP) T2 mapping. Patch-based denoising was optimized in patients without devices and impact on T2 precision and accuracy was assessed. Phantom studies included Bland-Altman and correlation analyses between the sequences. In-vivo performance was assessed through global and segmental T2 quantification, coefficient of variation (COV), artifact scoring, and edema detection. ANOVA with Bonferroni correction and pairwise testing were used for statistical comparisons.

**Results:**

In subjects without devices, wideband and conventional GRE T2 mapping yielded comparable T2 values (P = 0.60). With ICDs, conventional GRE T2 mapping underestimated global T2 by 16% (P<0.001) and increased segmental COV up to 30%. In contrast, wideband GRE T2 mapping provided accurate T2 values (P = 0.56) and preserved edema detection, showing relative T2 elevations of 44% comparable to bSSFP. Patch-based denoising significantly improved precision (P = 0.006) without biasing mean values (P = 0.999). Results were consistent across phantom, volunteer, patient, and animal experiments, including animal ex-vivo histology confirmation.

**Conclusion:**

Wideband GRE T2 mapping substantially reduced device-related artifacts, provided accurate T2 values, and allowed edema detection, offering a clinically feasible solution for patients with cardiac implants in this initial study.

## Graphical abstract

**Figure:**
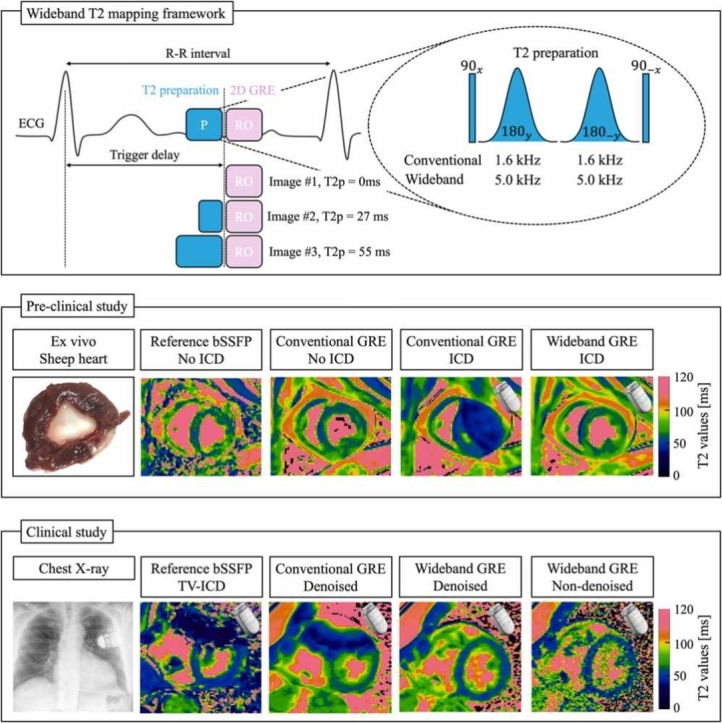

## 1 Introduction

According to data published in 2023 from the European Society of Cardiology, the average number of implantable cardioverter defibrillator (ICD) implantations per million people is estimated to be around 200–300 per million for the year 2019 [1]. Around 50%–75% of ICD patients are estimated to require cardiovascular magnetic resonance (CMR) imaging during their lifetime for follow-up imaging, such as diagnosis or treatment monitoring [2]. Accurate and non-invasive characterization of myocardial tissue is therefore critical to improve the risk stratification and management of this patient population.

Myocardial T2 mapping has emerged as a valuable tool for assessing myocardial tissue properties non-invasively, enabling quantitative measurement of myocardial tissue edema. T2 relaxation times are sensitive to changes in myocardial composition and provide insights into inflammatory processes, acute injury, and other pathological conditions.

In patients with ICDs, the assessment of myocardial tissue characteristics with CMR poses unique challenges. Some paramagnetic components of ICDs may create local field inhomogeneities and resonance frequency offsets around the device, often resulting in severe image artifacts that limit the diagnostic utility of conventional CMR. CMR techniques, including artifact reduction strategies, such as the use of gradient recalled echo (GRE) readout, and novel pulse sequences, have made CMR more feasible in ICD patients, opening new avenues for diagnostic and prognostic research [3]. In 2014, Rashid et al. first introduced the concept of wideband bright-blood phase-sensitive inversion recovery (PSIR) late gadolinium enhancement (LGE) imaging [4]. They proposed to broaden the spectral bandwidth of the inversion recovery pulse to 3.8 kHz to properly invert spins within the myocardium and reduce device-related hyperintensity artifacts. Gut et al. recently extended the wideband concept to black-blood LGE imaging to improve the identification of myocardial scars, which is often challenging with conventional and wideband bright-blood PSIR LGE imaging due to poor scar-blood contrast in ICD patients [5], [6]. In their wideband black-blood LGE imaging sequence, they used a wideband inversion pulse as proposed by Rashid et al. [4], followed by a wideband T2 preparation ($90_{x}-\;180_{y}-\;180_{-y}-90_{-y}$) [7] in which they broadened the spectral bandwidth of the adiabatic hyperbolic secant refocusing pulses to 5.0 kHz. Further advancements have been made for fibrosis assessment, with the works of Shao et al. [8] and Hong et al. [9] proposing wideband T1 mapping techniques specifically designed for ICD patients. While wideband approaches have improved LGE and T1 mapping in patients with ICDs, T2 mapping remains largely unexplored in this context [10]. Given the clinical importance of assessing myocardial inflammation and the challenges associated with T2 mapping in this growing population of patients with ICDs, wideband was extended to T2 mapping in this study using adiabatic T2 preparation, previously developed for wideband black-blood LGE imaging [5].

The purpose of this work was to implement a wideband T2 preparation in a myocardial T2 mapping sequence and to study the feasibility of wideband T2 mapping in the presence of implantable cardiac devices. The proposed wideband T2 mapping with a GRE readout was tested and compared against conventional GRE T2 mapping at 1.5T in a cardiac phantom, in healthy subjects both with and without an ICD placed on their chests, in patients with ICDs or pacemakers, and in animals with and without an external ICD.

## 2 Methods

All patients provided informed consent for participation in this study.

All animal experiments were performed following the guidelines from Directive 2010/63/EU of the European Parliament on the protection of animals used for scientific purposes. Protocols applied to sheep were approved by the local ethical committee (CEEA50) at the University of Bordeaux and by the French Government.

### 2.1 Sequence design

**Fig. 1:**
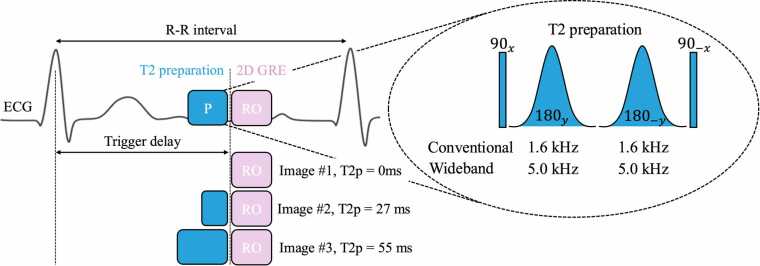
Conventional and wideband myocardial T2 mapping framework with a GRE readout. Three wideband T2-prepared single-shot images are acquired sequentially with increasing T2p duration (no preparation, 27 ms, and 55 ms), with two recovery heartbeats in-between. A two-parameter exponential fitting model is then employed to generate the T2 maps. *GRE* gradient-recalled echo, *T2p* T2 preparation

A two-dimensional, seven-heartbeat, breath-held, electrocardiogram (ECG)-triggered, single-shot T2 mapping sequence was implemented with wideband radiofrequency (RF) pulses. Three wideband T2 preparation echo times (TE = 0, 27, 55 ms) were acquired with two recovery heartbeats in between to allow magnetization recovery (Fig. 1) [11]. A spoiling gradient was then applied to remove any residual transverse magnetization. The adiabatic T2 preparation, relatively insensitive to B0 and B1 field inhomogeneities, consisted of a $90_{x}$ tip-down RF pulse, two adiabatic hyperbolic secant refocusing RF pulses with alternating phase $\left(180_{y}\;\mathrm{and}\;180_{-y}\right)$ and a duration of 12.8 ms, and a $90_{-y}$ tip-up RF pulse [7]. The spectral bandwidth of the refocusing pulses in the wideband T2 preparation was increased from 1.6 kHz to 5.0 kHz [5], [12]. To generate the different T2 preparation times, the delay between the two refocusing pulses was modified accordingly. A spoiled GRE readout was used to avoid the associated balanced steady-state free-precession (bSSFP) banding artifacts as seen on images acquired with conventional and wideband T2 mapping sequences [3]. For comparison, the clinical reference conventional bSSFP T2 mapping sequence with three T2 preparation echo times (TE = 0, 27, 55 ms) and two recovery heartbeats in between was acquired in healthy volunteers, patients, and an animal. Patients’ characteristics are provided in Table 1.

**Table 1:** Patient characteristics

Characteristics	Study 1	Study 2	Study 3
Number of participants	12	13	6
Female gender	6 (50)	6 (46)	1 (17)
Age, years	47.0 [32.5–68.3]	44.0 [33.0–68.0]	63.0 [61.0–68.0]
*Cardiomyopathy*			
Acute ischemic	1 (8)	1 (8)	0
Chronic ischemic	1 (8)	1 (8)	1 (17)
DCM	1 (8)	1 (8)	2 (33)
Myocarditis	4 (33)	5 (39)	1 (17)
Sarcoidosis	2 (17)	2 (15)	0
Auto-immune disease	1 (8)	1 (8)	0
Desminopathy	1 (8)	1 (8)	0
ARVC	0	0	1 (17)
Aortic regurgitation	0	0	1 (17)
Unknown origin	1 (8)	1 (8)	1 (17)
*Cardiac function*			
LVEF (%)	60.5 [58.75–62.5]	61.0 [59.0–61.0]	43.5 [21.8–49.5]
LVEF impairment (LVEF ≤ 40%)	1 (8)	1 (8)	3 (50)
LVEDVi (mL/m^2^)	75.5 [69.0–105.3]	78.0 [71.0–112.0]	96.0 [77.0–97.0]
LVESVi (mL/m^2^)	56.0 [46.0–73.3]	56.0 [47.0–80.0]	50.5 [41.0–87.3]
RVEF (%)	60.5 [58.8–65.3]	60.0 [58.0–65.0]	40.0 [28.0–55.0]
RVEF impairment (LVEF ≤ 40%)	0	0	3 (50)
RVEDVi (mL/m^2^)	73.5 [62.5–98.8]	78.0 [64.0–101.0]	126.0 [95.0–132.0]
RVESVi (mL/m^2^)	59.0 [43.3–74.0]	62.0 [46.0–80.0]	48.5 [40.3–65.5]
*Implantable cardiac devices*			
Pacemaker	0	0	2 (33)
TV-ICD	0	0	4 (67)
S-ICD	0	0	1 (17)

Values are given in mean ± standard deviation or median [interquartile range]. Percentages in brackets represent the percentage of the corresponding parameter for all the participants

*ARVC* arrhythmogenic right ventricular cardiomyopathy, *DCM* dilated cardiomyopathy, *ICD* implantable cardioverter defibrillator, *LVEDVi* indexed left ventricular end-diastolic volume, *LVEF* left ventricular ejection fraction, *LVESVi* indexed left ventricular end-systolic volume, *RVEDVi* right ventricular end-diastolic volume, *RVEF* right ventricular ejection fraction, *RVESVi* right ventricular end-systolic volume, *S-ICD* subcutaneous ICD, *TV-ICD* transvenous ICD

Study 1: HD-PROST parameters optimization in patients with myocardial inflammation and without devices. Study 2: Detection of absence or presence of myocardial inflammation in patients without devices. Study 3: Reduction of device-related artifacts in patients with devices

GRE T2 mapping with conventional T2 preparation, GRE T2 mapping with wideband T2 preparation and bSSFP T2 mapping with conventional T2 preparation were applied at 1.5T. Acquisitions parameters are provided in Table 2. T2 maps were reconstructed inline using a two-parameter (M0 and T2) fitting model [11].

**Table 2:** Acquisition parameters of reference conventional bSSFP T2 mapping, conventional GRE T2 mapping, and wideband GRE T2 mapping sequences

Parameters	Reference T2 mapping	Proposed T2 mapping
Application	–	Phantom
	Healthy volunteers	Healthy volunteers
	Patients	Patients
	Animal	Animal
Readout	2D bSSFP	2D GRE
Field of view (mm)	360 × 289	360 × 289
Resolution (mm)	1.9 × 1.9 × 8.0	1.9 × 1.9 × 8.0 1.4 × 1.4 × 8.0
Repetition time (ms)	2.49	2.91
Echo time (ms)	1.06	1.17
Window duration (ms)	107	197
Flip angle (°)	70	15
Phase oversampling (%)	0	0
RF pulse mode	Fast	Fast
Gradient mode	Fast	Fast
Parallel imaging	GRAPPA 2	GRAPPA 2
Phase partial Fourier	6/8	7/8
Asymmetric echo	Weak	Strong
Bandwidth (Hz/pixel)	1184	1221
Number of excitations	3	3
Number of slice positions	3	1–3
Recovery heartbeat (RR)	3	2
Acquisition time	9 heartbeats	7 heartbeats
Free-breathing	No	No
T2 preparation duration (ms)	0, 25, 55	0, 27, 55
Spectral bandwidth of the T2 preparation (kHz)	Conventional: 1.6 Wideband: 5.0	Conventional: 1.6 Wideband: 5.0

*bSSFP* balanced steady-state free-precession, *ECG* Electrocardiogram, *GRAPPA* generalized autocalibrating partially parallel acquisitions, *GRE* gradient recalled echo, *n/a* not applicable

For the proposed T2 mapping sequence, a resolution of 1.9 mm × 1.9 mm × 8.0 mm matching that of the reference conventional bSSFP T2 mapping sequence was used only for the healthy volunteer study 1: wideband T2 values accuracy in the absence of ICD

### 2.2 Denoising

High-dimensionality undersampled patch-based reconstruction PROST) was applied on the multi-contrast MR images prior to fitting [13]. HD-PROST works in three steps as follows: (1) Patch extraction and similarity grouping. For each spatial location in the image domain, a small patch (e.g., N×N voxels) is extracted across all contrast dimensions, in our case, three T2-prepared images. To exploit non-local redundancy, a set of similar patches, based on a similarity metric, is identified around each pixel located within a predefined fixed local window. The similar patches are then aggregated to form a three-dimensional tensor comprising spatial and contrast dimensions. (2) Low-rank tensor regularization. Once similar patches are grouped into a three-dimensional tensor, low-rank approximation is performed to keep only the most important features in the patch group and discard small variations that are likely to be noise or artifacts. A low-rank constraint, called regularization term, is imposed on each tensor to identify and remove inherent structural and contrast redundancies. (3) Denoised patch creation. The denoised tensor is then used to reconstruct the corresponding denoised patches. Steps (1) to (3) are applied across the entire image using a sliding window approach, ensuring that every patch location is processed. Since a given patch can appear in multiple overlapping groups during step 1, the final denoised multi-contrast images are generated by averaging the multiple estimates for each patch position. This averaging helps to further reduce noise and improve image consistency.

To perform HD-PROST, several parameters must be initialized. The radius of the fixed local window used to find similar areas around each pixel was set to 20, and the sliding search window was set to 200. The regularization term for the low-rank constraint and the patch size were optimized in a patient dataset without cardiac implants (see Section 2.5). The following values were initialized in the optimization process: regularization term = 4, 5, 6, 7, 8, 9, 10 and patch size = 5, 7.

### 2.3 Phantom study

A phantom study was carried out using the T1MES phantom [14], [15] on a 1.5T research MRI (MAGNETOM Aera, Siemens Healthineers, Erlangen, Germany) with a 32-channel spine coil and a dedicated 18-channel body coil to evaluate the T2 values obtained with the proposed wideband GRE T2 mapping sequence against the conventional GRE T2 mapping sequence. An ECG was simulated with a heart rate of 60 beats/minute. To evaluate the device-related artifact reduction performance using wideband GRE T2 mapping, an ICD (St. Jude, Sylmar, California, Quadra Assura CD3367–40Q) was placed 6 cm from the top of the phantom with cushions in between.

Global mean T2 values and standard deviations were measured for each of the nine tubes of T1MES. A Bland-Altman analysis was performed to compare the T2 values between the conventional and wideband GRE T2 mapping methods for each of the nine tubes, in the presence and absence of an ICD, and correlation coefficients were calculated.

### 2.4 Healthy volunteer study

Two studies in healthy volunteers were carried out on a 1.5T research MRI (MAGNETOM Avanto, Siemens Healthineers) with a 32-channel spine coil and a dedicated 18-channel anterior body coil. Three short-axis slices covering the heart at basal, mid-ventricular, and apical levels were acquired during mid-diastole and in breath-hold for the two datasets.

#### 2.4.1 Study 1: Reference T2 values comparison

To compare the T2 values obtained with conventional GRE T2 mapping and wideband GRE T2 mapping to the reference conventional bSSFP T2 mapping, these three sequences were acquired in a first dataset of six healthy volunteers (4 male, 31.5 [28.5–34.5] year-old). The same in-plane resolution of 1.9 mm × 1.9 mm was used for all the sequences.

T2 values were extracted in four American Heart Association (AHA) segments, anterior, septal, inferior, and lateral, for the three slices obtained with each sequence. A three-way ANOVA test and pairwise comparisons with Bonferroni correction were performed to assess the effect of the used sequence, slice position (basal, mid-ventricular, apical), and segment on the T2 values. A P-value <0.05 was considered statistically significant.

#### 2.4.2 Study 2: ICD-associated artifact

To assess the in-vivo performance of the proposed wideband GRE T2 mapping for ICD-associated artifact reduction and to compare T2 values in the presence and absence of an ICD, conventional GRE and wideband GRE T2 mapping were applied in a second dataset of eight healthy volunteers (6 male, 24.5 [23.0–27.0] year-old). To simulate device-induced image artifacts, an ICD (St. Jude Medical, Quadra Assura, model CD3265–40Q) was placed on the left clavicle approximately 10 cm from the heart. In total, four acquisitions were performed for each volunteer: conventional and wideband GRE T2 mapping without ICD, conventional and wideband GRE T2 mapping with ICD. An in-plane resolution of 1.4 mm × 1.4 mm was used for the conventional and wideband GRE T2 mapping sequences to further reduce ICD-associated artifacts [3].

T2 values were extracted for the 16 AHA segments. Repeated measurements ANOVA and pairwise comparisons with Bonferroni correction were performed to compare T2 values between sequences. The coefficient of variation (COV) for each of the 16 AHA segments was calculated as the ratio of the standard deviation to the mean T2 value.

### 2.5 Patient study

All patients were referred for cardiac MRI on 1.5T system at the University Hospital of Bordeaux in France (MAGNETOM Aera, Siemens Healthineers) and the University Hospital of Lausanne in Switzerland (MAGNETOM Sola, Siemens Healthineers) for clinical purposes independent of this study. In the two centers, patients were scanned with a 32-channel spine coil and a dedicated 18-channel anterior body coil. All patients were imaged with the proposed wideband GRE T2 mapping sequence, the conventional GRE T2 mapping sequence, and the clinical reference conventional bSSFP T2 mapping sequence. All images were acquired in mid-diastole and during a breath-hold. Specific absorption rate was maintained below 2 W/kg. Three patient studies were carried out. Patients’ characteristics are provided in Table 2.

#### 2.5.1 Study 1: HD-PROST parameter optimization

Twelve patients with known cardiomyopathy and without implanted cardiac devices were included in this study. Included patients were not allowed to have diffuse T2 elevation in order to have a myocardial region of interest (ROI) unaffected by T2 elevation, hereafter referred to as remote ROI. A total of 14 HD-PROST parameters were tested, combining a patch size of 5 or 7 and a regularization term of 4 to 10, on the multi-contrast T2-weighted images obtained with wideband GRE T2 mapping. For each patient, one remote ROI was contoured on the original (not denoised) T2 map in a region were no increased T2 values was present, known from the reference conventional bSSFP T2 maps. The same ROI was then applied on the denoised images obtained for each HD-PROST parameter combinations. Mean T2 values and standard deviations in the ROI were extracted from the original and denoised images.

Accuracy and precision of the denoised T2 values over all patients were assessed by:

Accuracy = mean ± SD of the mean T2 values over all patients

Precision = mean ± SD of the standard deviations of the T2 values over all patients

Repeated one-way analysis of variance (ANOVA) tests and pairwise comparisons with Bonferroni correction were performed to evaluate the effect of the used HD-PROST parameters on T2 values. After assessing accuracy and precision, and performing statistical analysis on T2, the best combination of a regularization term and patch size was identified and used for the denoised maps in all sections.

#### 2.5.2 Study 2: Detection of absence or presence of myocardial inflammation without ICD

Thirteen patients with known cardiomyopathy, not necessarily presenting with myocardial inflammation, and without implanted cardiac devices, were included in this study to evaluate the performance of wideband GRE T2 mapping and conventional GRE T2 mapping in identifying the absence or elevation of T2 values observed on clinical conventional bSSFP images. One short-axis slice was acquired for all sequences.

For each patient and each sequence, one ROI was contoured in a region of elevated T2 values (edema ROI), if present, and another one in a remote myocardial region (remote ROI) on the denoised T2 maps. Same ROIs were applied to the original T2 maps. Mean T2 values and standard deviations in each ROI were extracted from the original and denoised T2 maps and were compared to the ground-truth conventional bSSFP T2 mapping using repeated measurements ANOVA and pairwise comparisons with Bonferroni correction.

#### 2.5.3 Study 3: Reduction of device-related artifacts

Seven patients with previously implanted cardiac devices (2× pacemaker, 5x ICD) who were not device-dependent were included in this study to assess the performance of wideband GRE T2 mapping in reducing device-related artifacts. One short-axis slice was acquired for all sequences. Reference conventional bSSFP T2 maps were not acquired in all patients, as in some cases, the MRI exam was interrupted due to severe artifacts, and therefore was not included in the statistical analyses. The devices were interrogated and reprogrammed before and after the MR scan [16], [17]. One expert radiologist graded the image artifact severity score with a 4-point Likert scale (1 = non-diagnostic, 2 = large artifacts, 3 = moderate artifacts, 4 = minimal artifacts).

### 2.6 Animal study

An animal study with a sheep (ovine) was conducted at LIRYC in Bordeaux, France. Ischemic scars were created in the left ventricle by progressively occluding the apical left anterior descending coronary artery and the medial left circumflex coronary artery, deploying an embolization coil pushed by a microcatheter to reduce blood flow. Reperfusion was performed after 90 min. Six weeks after occlusion and just before performing CMR, multifocal endocardial ablation was performed to induce edema.

Before occlusion and six weeks after occlusion, cardiac MRI was performed with conventional and wideband GRE T2 mapping and reference conventional bSSFP T2 mapping on a 1.5T research MRI (MAGNETOM Aera, Siemens Healthineers) with a 32-channel spine coil and an 18-channel cardiac coil. During MRI, the animal was intubated, ventilated and under general anesthesia. The three sequences were applied with and without an ICD (St. Jude Medical, Sylmar, California, USA, QUADRA ASSURA, model CD3265–40Q), for a total of six acquisitions. In case of ICD, the generator was placed on the left shoulder of the sheep approximately 10 cm away from the heart. Three short-axis slices covering the heart at basal, mid-ventricular, and apical levels were acquired during mid-diastole in breath-hold. Ventilation was temporarily suspended when breath-holds were needed during scans. Specific absorption rate was maintained below 2 W/kg.

Mean T2 values and standard deviations were extracted from the 16 AHA segments for all sequences before and after ischemia.

## 3 Results

### 3.1 Phantom study

**Fig. 2:**
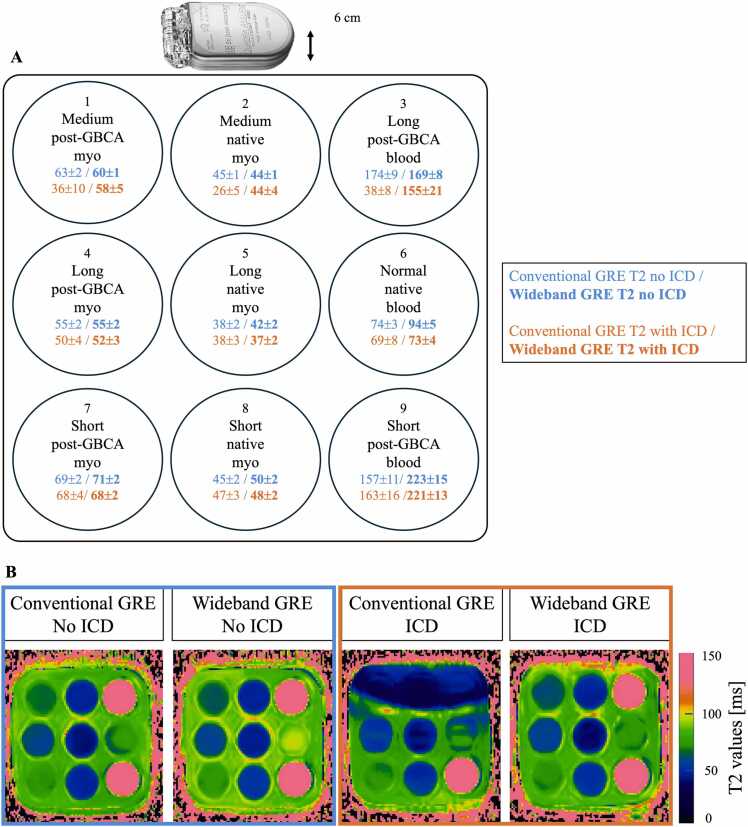
Results for the phantom study. (**A**) Mean measured T2 values in the nine tubes of the T1MES phantom using standard and wideband GRE T2 mapping without an ICD (blue) and with an ICD (orange). (**B)** Corresponding T2 maps in the absence (blue) and presence (orange) of an ICD. *GBCA* gadolinium-based contrast agent, *ICD* implantable cardioverter defibrillator, *GRE* gradient-recalled echo

Measured global mean T2 values in the nine tubes using conventional and wideband GRE T2 mapping with and without ICD are shown in Fig. 2**A**, and corresponding T2 maps are shown in Fig. 2**B**. Bland-Altman analysis (Supplementary Fig. S1) showed that T2 values obtained with conventional GRE T2 mapping in absence of ICD, and wideband GRE T2 mapping in absence and presence of ICD had excellent correlation between all these three measurements (r>0.9), except for long T2 values representing blood (tubes 3 and 9).

### 3.2 Healthy volunteer study

#### 3.2.1 Study 1: Reference T2 values comparison

Segmental T2 values are reported in Table 3 **Study1**. From the three-way ANOVA test, it was shown that the sequence used had an effect on T2 measurements (P = 0.039). Pairwise comparisons showed that the sequence used affected the anterior (P = 0.008), lateral (P = 0.031), and septal (0.008) T2 values. However, the sequence used and the segment of interest together did not have an effect on T2 measurements (P = 0.760). Moreover, the segment of interest did not have an effect on T2 measurements (P = 0.548), neither the slice position (P = 0.111).

**Table 3:** Segmental and global T2 values obtained in healthy volunteers

Sequence	Segmental mean T2 values (ms)				Global mean T2 values (ms)
	Anterior	Inferior	Lateral	Septal	
*Study 1: Reference T2 values comparison*					
Reference bSSFP	48.8±4.3	48.6±3.4	48.2±3.0	48.3±3.4	48.5±3.5
Conventional GRE P-value wrt. bSSFP	42.4±4.4 0.084	43.5±4.2 0.579	43.1±6.9 0.174	42.5±5.2 0.114	42.9±5.2 <0.001 (*)
Wideband GRE P-value wrt. bSSFP P-value wrt. Conv. GRE	43.0±5.1 0.096 0.999	43.0±5.6 0.133 0.621	41.6±5.1 0.210 0.999	41.3±5.1 0.077 0.828	42.2±5.2 <0.001 (*) 0.660
*Study 2: ICD-associated artifact reduction*					
Conventional GRE T2 mapping, without ICD	42.9±2.7	44.3±2.6	43.1±1.5	43.5±1.9	43.5±2.2
Conventional GRE T2 mapping, with ICD	32.2±5.2	39.5±3.7	36.9±5.9	37.1±6.8	36.4±5.9
P-value	0.009 (*)	0.076	0.161	0.163	<0.001 (*)
Wideband GRE T2 mapping, without ICD	42.2±2.3	43.5±2.4	42.0±1.0	43.8±1.6	42.9±2.0
Wideband GRE T2 mapping, with ICD	43.7±2.7	44.3±2.1	42.6±1.3	45.1±1.8	43.9±2.1
P-value	0.167	0.999	0.999	0.999	0.240

Values are given in mean ± standard deviation. A significant P-value is indicated with (*).

*bSSFP* balanced steady-state free-precession, *GRE* gradient recalled echo, *n/a* not applicable, *ICD* implantable cardioverter defibrillator, *wrt.* with respect to

Global mean T2 values were significantly different between conventional bSSFP and conventional GRE T2 mapping (P<0.001) and between conventional bSSFP and wideband GRE T2 mapping (P<0.001). Global mean T2 values were on the other hand, comparable between conventional GRE and wideband GRE T2 mapping (P = 0.66).

#### 3.2.2 Study 2: ICD-associated artifact reduction

**Fig. 3:**
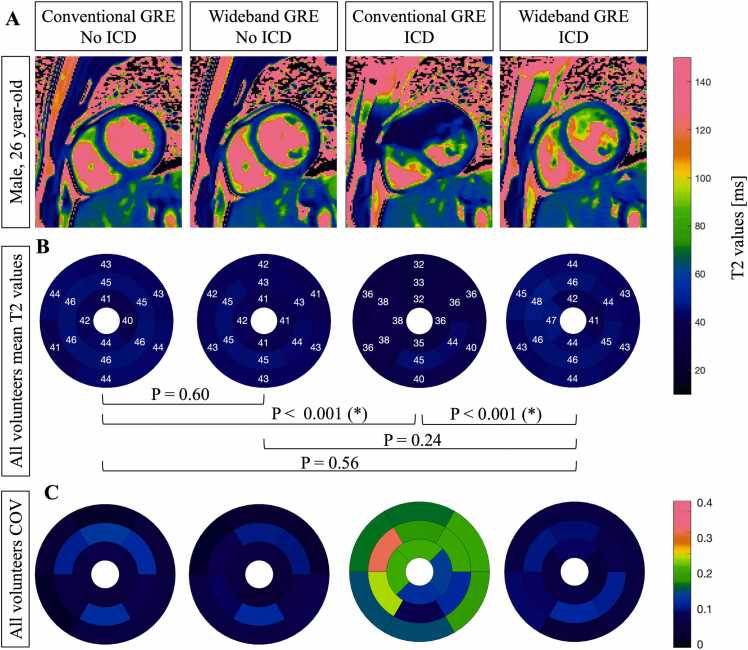
Results for the healthy volunteer study. (**A**) Example of conventional and wideband GRE T2 mapping with and without an external ICD in a 27-year-old male healthy volunteer (**B**) Segmental analysis of mean T2 values in the eight healthy volunteers using conventional and wideband GRE T2 mapping in the absence and presence of ICD. (**C**) Segmental analysis of the COVs of T2 values in the eight healthy volunteers using conventional and wideband GRE T2 mapping in absence and presence of ICD. Note: A significant P-value is indicated with (*). *COV* coefficient of variations, *ICD* implantable cardioverter defibrillator, *GRE* gradient-recalled echo

An example of T2 maps obtained in a healthy volunteer with conventional and wideband GRE T2 mapping with and without an external ICD is shown in Fig. 3**A**. Fig. 3**B** shows 16-AHA segmental mean T2 values in all eigth healthy volunteers obtained with conventional and wideband GRE T2 mapping in presence and absence of ICD. Segmental and global mean T2 values for the anterior, inferior, lateral, and anterior segments are reported in Table 3 **Study 2**. Global mean T2 values measured without an ICD were comparable between the conventional (43.5 ± 2.2 ms) and wideband technologies (42.9 ± 2.0 ms) (P = 0.60). In the presence of an ICD, global mean T2 values were significantly reduced with the conventional sequence (36.4 ± 5.9 ms), with the most affected segements being the anterior segments (P<0.01), compared to the wideband sequence (43.9 ± 2.1 ms) (P<0.001). Using a wideband T2 preparation, ICD-related artifacts affecting the myocardium were reduced, allowing for accurate T2 values (43.9 ± 2.1 ms), consistent with conventional T2 values observed in the absence of an ICD (P = 0.56).

Fig. 3C shows 16-AHA segmental COVs in all eigth healthy volunteers for all measurements. For wideband T2 mapping, with and without an ICD, and for conventional T2 mapping without an ICD, the COV in each segment was below 13%, indicating low variability. In contrast, COVs for conventional T2 mapping in presence of ICD were higher in all segments, with the highest variability in the mid anteroseptal segment being of 30%.

### 3.3 Patient study

Patient characteristics can be found in Table 2.

#### 3.3.1 Study 1: HD-PROST parameters optimization without ICD

The precision and accuracy of each combination are shown in Supplementary Fig. S2. Repeated measures one-way ANOVA showed that the HD-PROST parameters used had an effect on the mean (P<0.001) and standard deviations (P<0.001) of remote myocardial T2 values. The results of pairwise comparisons between each combination of HD-PROST parameters and the original map on mean and standard deviations of T2 values are presented in Supplementary Table **T1**. Based on these results and visualization of the denoised T2 maps with the different parameter combinations, the combination of a regularization term of 4 and a patch size of 7 was selected and used for subsequent studies. An example before and after denoising spoiled GRE T2-weighted images using a regularization term of 4 and a patch size of 7 and resulting calculated T2 map in a patient with myocardial infarction with non-obstructive coronary arteries is shown in Supplementary Fig. S3.

#### 3.3.2 Study 2: Detection of absence or presence of myocardial inflammation without ICD

**Fig. 4:**
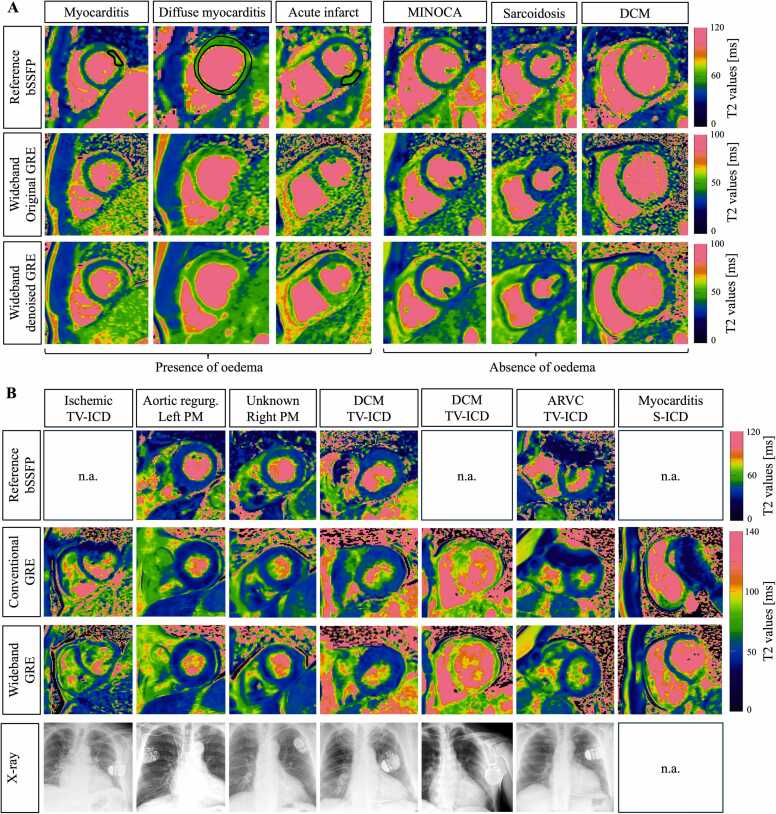
Results for the patient study. (**A**) Patients without implantable devices. Columns 1–3: Example of presence of edema in reference bSSFP and wideband denoised GRE T2 mapping in patients with myocarditis, diffuse myocarditis, and acute infarct. Columns 4–6: Example of absence of edema in reference conventional bSSFP and wideband denoised GRE T2 mapping in patients with MINOCA, pulmonary sarcoidosis, and DCM. (**B**) Patients with cardiac implantable devices. T2 maps with TV-ICDs (transvenous implantable cardioverter defibrillators) and S-ICD (subcutaneous) are more affected by image artifacts than those with PMs. *ARVC* arrhythmogenic right ventricular cardiomyopathy, *n.a.* not available, *bSSFP* balanced steady-state free-precession, *GRE* gradient recalled echo, *MINOCA* myocardial infarction with non-obstructive coronary arteries, *DCM* dilated cardiomyopathy, *PM* pacemakers

Seven patients (54% (7/13)) showed T2 elevation in clinical conventional bSSFP T2 maps. Fig. 4**A** shows examples of the presence of edema, as well as examples of the absence of edema, in reference to conventional bSSFP T2 maps and denoised wideband GRE T2 maps.

##### 3.3.2.1. Remote

Mean T2 values in remote myocardium in the thirteen patients for each sequence are reported in Table 4. One-way ANOVA test showed that the sequence used had an effect on the T2 measurements (P< 0.001). Mean T2 values for original conventional (34.6 ± 3.63 ms) and denoised conventional (35.2 ± 3.41 ms) GRE T2 maps were similar (P = 0.99), as well as for original wideband (33.7 ± 2.57 ms) and denoised wideband (34.5 ± 2.20 ms) GRE T2 maps (P = 0.48). Moreover, no difference in T2 values was found between original conventional and original wideband T2 values (P = 0.99), likewise for denoised conventional and denoised wideband T2 values (P = 0.99). Compared to conventional bSSFP T2 values (44.1 ± 1.55 ms), significant differences in T2 measurements were found with original conventional GRE (P<0.001), denoised conventional GRE (P<0.001), wideband GRE (P<0.001), and denoised wideband GRE (P<0.001).

**Table 4:** Results for the patient study 2: Detection of absence or presence of myocardial inflammation without ICD

Sequence	Remote mean T2 values (ms) N=13	Edema mean T2 values (ms) N=7	P-value
Reference conventional bSSFP	44.1 ± 1.6	60.5 ± 3.8	<0.001 (*)
*Conventional GRE, Original* p-value wrt. bSSFP p-value wrt. wideband original p-value wrt. wideband denoised	34.6 ± 3.6 <0.001 (*) 0.99 0.99	49.0 ± 2.2 0.008 (*) 0.99 0.99	<0.001 (*)
*Conventional GRE, Denoised* p-value wrt. bSSFP p-value wrt. conventional original p-value wrt. wideband original p-value wrt. wideband denoised	35.2 ± 3.4 <0.001 (*) 0.999 0.99 0.99	48.2 ± 1.9 0.003 (*) 0.181 0.328 0.99	<0.001 (*)
*Wideband GRE, Original* p-value wrt. bSSFP	33.7 ± 2.6 <0.001 (*)	50.3 ± 1.1 0.007 (*)	<0.001 (*)
*Wideband GRE, Denoised* p-value wrt. bSSFP p-value wrt. wideband denoised	34.5 ± 2.2 <0.001 (*) 0.48	49.5 ± 1.3 0.006 (*) 0.99	<0.001 (*)

Values are given in mean ± standard deviation. A significant P-value is indicated with (*)

*bSSFP* balanced steady-state free-precession, *GRE* gradient recalled echo, *n/a* not applicable, *wrt.* with respect to

##### 3.3.2.2. Edema

Mean T2 values in edema in the seven patients for each sequence are reported in Table 4. Two-way ANOVA showed that the sequence used and the ROI considered (either remote or edema) had both an effect on the T2 measurements (both P<0.001). Edema mean T2 values for original conventional (49.0 ± 2.22 ms) and denoised conventional (48.2 ± 1.88 ms) GRE T2 maps were similar (P = 0.181), as well as for original wideband (50.3 ± 1.08 ms) and denoised wideband (49.5 ± 1.30 ms) GRE T2 maps (P = 0.99). Moreover, no difference in T2 values was found between original conventional and original wideband T2 values (P = 0.99), likewise for denoised conventional and denoised wideband T2 values (P = 0.99). Compared to conventional bSSFP T2 values (60.5 ± 3.76 ms), significant differences in T2 values were found with original conventional GRE (P = 0.008), denoised conventional GRE (P = 0.003), wideband GRE (P = 0.007), and denoised wideband GRE (P = 0.006).

Compared to mean T2 values in remote myocardial region, mean T2 values in edema were significantly higher for conventional bSSFP (P<0.001), original conventional GRE (P<0.001), denoised conventional GRE (P<0.001), original wideband GRE (P<0.001), and denoised wideband GRE (P<0.001) T2 mapping.

#### 3.3.3. Study 3: Reduction of device-related artifacts

An example of T2-weighted images obtained in a patient with a TV-ICD for the different T2 preparation times and for conventional bSSFP, conventional spoiled GRE, and wideband spoiled GRE is shown in Supplementary Fig. S4.

Reference conventional bSSFP, if available, conventional GRE and wideband GRE T2 mapping in all patients are shown in Fig. 4**B**. In two patients, reference bSSFP T2 maps were not available. In patients with ICDs, reference conventional bSSFP T2 maps were more affected (score = 2.6 ± 1.8) than conventional GRE T2 maps (score = 3.0 ± 1.9) due to the banding artifacts. Wideband GRE T2 maps, on the other hand, were less artefacted (score = 4.3 ± 1.0) than conventional GRE T2 maps. In the patient with a subcutaneous ICD (S-ICD), image artifacts were drastically reduced, but the inferolateral and lateral segements were still artefacted. In a patient with TV-ICD, wideband GRE T2 map showed reduced artifacts compared to the conventional T2 GRE map, but the anterior segment still contained artifacts. In patients with pacemakers, the myocardium was not affected by device artifacts in all sequences.

### 3.4 Animal study

**Fig. 5:**
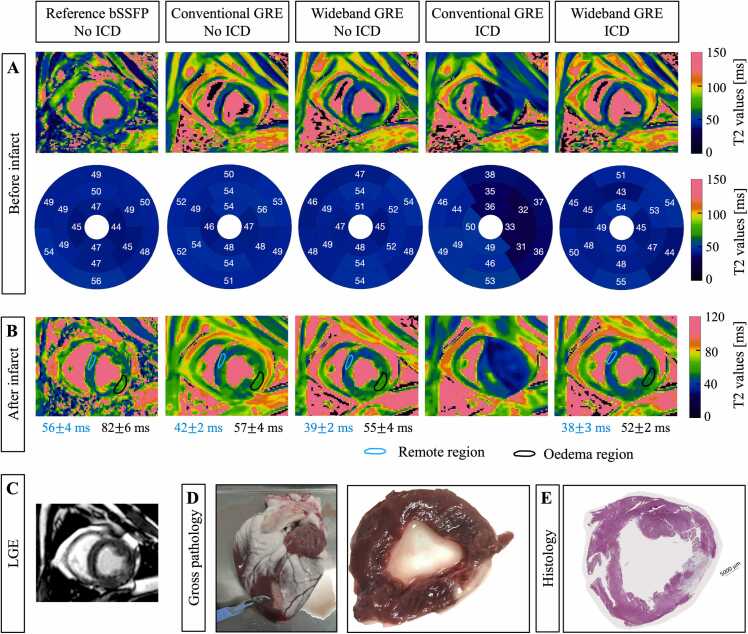
Results of the animal study. T2 maps obtained with reference conventional T2-prepared balanced steady-state free-precession (bSSFP), conventional T2-prepared gradient-recalled echo (GRE) and wideband T2-prepared GRE, with and without an implantable cardioverter defibrillator (ICD), before infarct (**A**) and after multifocal ablation (**B**). (**C**) Late gadolinium enhancement (LGE) images show the infarct six weeks post-occlusion. (**D**) Ex-vivo gross pathology and (**E**) histology confirms the presence of chronic infarct

In the case before infarction (Fig. 5**A**), bullseye plots showed similar T2 values in all segments between conventional bSSFP, conventional GRE, and wideband GRE T2 mapping when no ICD was placed. When the ICD was attached to the animal, the T2 values obtained with conventional GRE T2 mapping in the anteroseptal, anterior, and anterolateral segments were decreased by more than 10 ms. Wideband GRE T2 mapping provided more accurate T2 values in these segments.

In the case after infarction (Fig. 5**B**), edema could be detected using all of the different sequences. Conventional GRE and wideband GRE T2 mapping in absence of ICD, and wideband GRE T2 mapping in presence of ICD provided a mean T2 value in remote region of 40 ± 2 ms and in edema of 55 ± 3 ms, with a relative increase of 38% compared to remote region, against conventional bSSFP with a mean T2 value in remote region of 56 ± 4 ms and in edema of 82 ± 6 ms, with a relative increase of 46% compared to remote region.

## 4 Discussion

Previous wideband implementations have primarily targeted LGE imaging and, more recently, T1 mapping for ICD patients. In this work, the concept of wideband was extended to adiabatic T2 mapping. Given the central role of T2 mapping in detecting myocardial inflammation, this technique may facilitate earlier diagnosis and better monitoring in conditions such as myocarditis, transplant rejection, and acute ischemia. In this study, we implemented and evaluated a wideband T2 preparation module within a GRE-based myocardial T2 mapping sequence and using HD-PROST denoising strategy to address the challenges of device-related image artifacts in patients with implantable cardiac devices. The proposed method was tested across phantom, healthy volunteers, patient, and animal studies, and compared against conventional GRE T2 mapping and reference conventional bSSFP T2 mapping. Advanced denoising strategy with HD-PROST was applied on native T2-weighted images prior to fitting to exploit the highly redundant information on a local and non-local scale, and the strong anatomical correlation between the different contrast images.

The phantom experiment showed that, in the absence of ICDs, conventional and wideband GRE T2 mapping yield comparable results. Moreover, when an ICD was placed, conventional GRE T2 mapping showed substantial T2 decrease in the vicinity of the device, whereas the wideband sequence preserved accurate T2 values, demonstrating effective mitigation of off-resonance effects caused by the device.

In volunteers without ICD placed, wideband GRE and conventional GRE T2 mapping yielded similar global T2 values of 42.9 ± 2.0 ms and 43.5 ± 2.2 ms, respectively, showing that no systematic bias was introduced with wideband modification. In contrast, both GRE-based sequences underestimated T2 values by 10% compared to reference conventional bSSFP T2 mapping. With an ICD present, conventional GRE T2 mapping underestimated T2 values in region of artifacts, particularly in anterolateral, anterior, and anteroseptal segments, with a 16% reduction in global T2 (36.4 ± 5.9 ms) and up to 30% coefficient of variation. Wideband GRE T2 mapping yielded accurate T2 values matching ICD-free measurements (P = 0.56), and reduced segmental variability by 50%.

In patients without devices, the parameter optimization for HD-PROST yielded a patch size of 7 and a regularization term of 4. This parameter combination reduced standard deviation in remote myocardium while preserving mean T2 values. Both wideband and conventional GRE T2 mapping differentiated edematous from remote myocardium, with results consistent before and after denoising. Denoising did not alter the T2 relative differences between conventional and wideband GRE T2 mapping and between remote and edema ROIs. Edematous regions showed an increase in T2 values of approximately 44% relative to their healthy remote region using wideband GRE T2 mapping, similar to reference conventional bSSFP T2 mapping and which is consistent with the literature [18]. However, T2 values obtained with wideband GRE (remote: 34.5 ± 2.2 ms, edema: 49.5 ± 1.3 ms) remained lower than reference conventional bSSFP (remote: 44.1 ± 1.6 ms, edema: 60.5 ± 3.8 ms) by approximately 10 ms in remote myocardium and 11 ms in edema. These results were consistent with those of the animal study, in which the edematous regions also showed an increase in T2 values of approximately 38% compared to their remote healthy region using wideband T2 GRE mapping.

In patients with implanted cardiac devices, wideband GRE T2 mapping reduced device-related artifacts compared to conventional GRE. This was especially evident in patients with ICDs, where anterior and septal myocardial segments were most prone to artifacts, with a T2 underestimation of about 12 ms. Although some residual artifacts remained in some cases when the ICD was close to the heart (S-ICD), delineation of anterior and septal LV segments were substantially improved.

## 5 Limitations

This study has several limitations. Sample sizes in the patient and animal studies were relatively small. Although our proposed method could detect T2 elevation in edema regions, further studies across more diverse cardiomyopathies and in more patients are needed. Also, large-scale studies in patients with ICDs and myocardial inflammation are warranted to validate the proposed sequence.

The proposed sequence was applied in breath-hold without motion correction. However, inadequate breath-holding can result in native image mis-registration, resulting in mapping errors and inaccurate T2 values [19]. The use of non-rigid motion correction on native images could improve the accuracy of T2 values and could be investigated in future studies.

Moreover, while wideband GRE T2 mapping drastically reduced artifacts masking the myocardium, complete artifact suppression was not always possible, particularly when the device was positioned very close to the myocardium. In future studies, a frequency offset could be applied to further reduce off-resonance artifacts [4].

Another limitation of this study was the observed differences in T2 values between GRE and bSSFP T2 mapping. To our knowledge, no evidence of a systematic decrease in T2-prepared GRE versus T2-prepared bSSFP in myocardium at 1.5T has been reported in the literature. In the proposed implementation of T2-prepared GRE mapping, a spoiled gradient echo readout was used to eliminate residual transverse magnetization. As a result, and in combination with limited recovery heartbeats, longitudinal magnetization is reduced compared to a bSSFP readout, and residual T1 weighting may be introduced, potentially causing underestimation of T2 values. Furthermore, the use of high B1 amplitude wideband adiabatic T2 preparation pulses may introduce additional rotating-frame (T2rho) relaxation during the preparation module. When combined with limited recovery between each echo time, these effects may further impact measured T2 values.

More investigations should be performed in the future for clinical translation, to establish spoiled GRE-specific thresholds for the diagnosis of inflammation.

Furthermore, in all sequences used in this work, a recovery of two heartbeats was defined between each echo. In case of fast and variable heartbeat, incomplete recovery of magnetization leads to an underestimation of T2 measurements. Future work could explore the application of wideband myocardial T2 mapping with longer recovery to avoid T1 weighting, or the use of a 90° saturation pulse prior to acquisition to avoid heart rate dependence. Finally, the proposed spoiled GRE T2-prepared mapping sequence used three native contrast images to calculate the T2 map. The use of spoiled GRE for image acquisition reduces the signal-to-noise ratio compared to bSSFP [12]. An additional average could be explored in order to acquire two contrast images to increase the signal-to-noise ratio before applying the HD-PROST denoising strategy, but the acquisition time will also increase. Also, other denoising strategies, including artificial intelligence, could also be explored in future studies [20].

## 6 Conclusion

This work demonstrated that wideband GRE T2 mapping can mitigate off-resonance frequency sensitivity of conventional T2-prepared GRE and provide accurate myocardial T2 quantification in the presence of ICDs and pacemakers. The proposed technique preserved sensitivity to myocardial edema detection, with relative T2 elevations comparable to those seen in conventional bSSFP T2 mapping. Application of HD-PROST denoising preserved the accuracy of T2 values and increased precision by selecting the appropriate parameters used in the denoising process, which allowed better myocardial visualization and allowed to distinguish edematous from normal myocardium. These findings indicate that wideband GRE T2 mapping combined with advanced denoising may enable more accurate myocardial T2 quantification with substantial artifact reduction in the presence of ICDs and pacemakers.

## Funding

This research was supported by funding from the French National Research Agency under grant agreements Equipex MUSIC ANR-11-EQPX-0030, ANR-22-CPJ2–0009-01, ANR-21-CE17–0034-01, Programme d’Investissements d’Avenir ANR-10-IAHU04-LIRYC, the French Federation of Cardiology (*Federation FranÇaise de Cardiologie*), and from the European Research Council (ERC) under the European Union's Horizon 2020 research and innovation program (grant agreement 101076351).

## Author contributions

**Pauline Gut: Writing – original draft, Validation, Methodology, Formal analysis,** Data curation. **Hubert Cochet:** Visualization, Project administration, Data curation. **Kustner Thomas:** Resources, Data curation. **Guido Caluori:** Visualization, Resources, Data curation. **Konstantinos Vlachos:** Visualization, Data curation. **Panagiotis Antiochos:** Visualization, Data curation. **Ambra Masi:** Data curation. **Juerg Schwitter:** Data curation. **Frederic Sacher:** Project administration. **Jais Pierre JaÏs:** Project administration. **Matthias Stuber:** Writing – review & editing, Supervision, Resources, Project administration, Investigation, Funding acquisition, Conceptualization. **AurÉlien Bustin:** Writing – review & editing, Supervision, Resources, Project administration, Methodology, Investigation, Funding acquisition, Data curation.

## Declaration of competing interests

The authors declare that they have no known competing financial interests or personal relationships that could have appeared to influence the work reported in this paper.

## Data Availability

The datasets used and/or analyzed during the current study are available from the corresponding author on reasonable request.

## Footnotes

Appendix A Supplementary data associated with this article can be found in the online version at doi:10.1016/j.jocmr.2026.102717.

## References

[1] Farkowski M.M., Scherr D., Boriani G., Kazakiewicz D., Haim M., Huculeci R. (2025).

[2] Kalin R., Stanton M.S. (2005). Current clinical issues for MRI scanning of pacemaker and defibrillator patients. Pacing and Clinical Electrophysiology.

[3] Gut P., Cochet H., Stuber M., Bustin A. (2024). Magnetic resonance myocardial imaging in patients with implantable cardiac devices: challenges, techniques, and clinical applications. Echocardiogr.

[4] Rashid S., Rapacchi S., Vaseghi M., Tung R., Shivkumar K., Finn J.P. (2014). Improved late gadolinium enhancement MR imaging for patients with implanted cardiac devices. Radiol Radiol Soc North Am.

[5] Gut P., Cochet H., Caluori G., El-Hamrani D., Constantin M., Vlachos K. (2024). Wideband black-blood late gadolinium enhancement imaging for improved myocardial scar assessment in patients with cardiac implantable electronic devices. Magn Reson Med.

[6] Gut P., Cochet H., Antiochos P., Caluori G., Durand B., Constantin M. (2024). Improved myocardial scar visualization using free-breathing motion-corrected wideband black-blood late gadolinium enhancement imaging in patients with implantable cardiac device. Diagn Inter Imaging.

[7] Nezafat R., Ouwerkerk R., Derbyshire A.J., Stuber M., McVeigh E.R. (2009). Spectrally selective B1-insensitive T2 magnetization preparation sequence. Magn Reson Med.

[8] Shao J., Rashid S., Renella P., Nguyen K.L., Hu P. (2017). Myocardial T1 mapping for patients with implanted cardiac devices using wideband inversion recovery spoiled gradient echo readout. Magn Reson Med.

[9] Hong K., Jeong E.K., Wall T.S., Drakos S.G., Kim D. (2015). Wideband arrhythmia-Insensitive-rapid (AIR) pulse sequence for cardiac T1 mapping without image artifacts induced by an implantable-cardioverter-defibrillator. Magn Reson Med.

[10] Pan Y., Liu Y., Varghese J., Simonetti O.P. (2020). Improved myocardial T2 mapping in the presence of a cardiac implanted electronic device. Proc 23rd Annu SCMR Sci Sess.

[11] Giri S., Chung Y.C., Merchant A., Mihai G., Rajagopalan S., Raman S.V. (2009). T2 quantification for improved detection of myocardial edema. J Cardiovasc Magn Reson.

[12] Gut P., Cochet H., Antiochos P., Caluori G., Durand B., Constantin M. (2025). Improved myocardial scar visualization using free-breathing motion-corrected wideband black-blood late gadolinium enhancement imaging in patients with implantable cardiac devices. Diagn Interv Imaging.

[13] Bustin A., Lima da Cruz G., Jaubert O., Lopez K., Botnar R.M., Prieto C. (2019). High-dimensionality undersampled patch-based reconstruction (HD-PROST) for accelerated multi-contrast MRI. Magn Reson Med.

[14] Captur G., Gatehouse P., Keenan K.E., Heslinga F.G., Bruehl R., Prothmann M. (2016). A medical device-grade T1 and ECV phantom for global T1 mapping quality assurance - the T1 mapping and ECV standardization in cardiovascular magnetic resonance (T1MES) program. Journal of Cardiovascular Magnetic Resonance.

[15] Captur G., Bhandari A., BrÜhl R., Ittermann B., Keenan K.E., Yang Y. (2020). mapping performance and measurement repeatability: results from the multi-national T 1 mapping standardization phantom program (T1MES). J Cardiovasc Magn Reson.

[16] Nazarian S., Roguin A., Zviman M.M., Lardo A.C., Dickfeld T.L., Calkins H. (2006). Clinical utility and safety of a protocol for noncardiac and cardiac magnetic resonance imaging of patients with permanent pacemakers and implantable-cardioverter defibrillators at 1.5 Tesla. Circulation.

[17] Nazarian S., Halperin H.R. (2009). How to perform magnetic resonance imaging on patients with implantable cardiac arrhythmia devices. Heart Rhythm.

[18] Van Heeswijk R.B., Feliciano H., Bongard C., Bonanno G., Coppo S., Lauriers N. (2012). Free-breathing 3 T magnetic resonance T2-mapping of the heart. JACC Cardiovasc Imaging Elsevier.

[19] O’Brien A.T., Gil K.E., Varghese J., Simonetti O.P. (2022). T2 mapping in myocardial disease: a comprehensive review. J Cardiovasc Magn Reson.

[20] Morales M.A., Manning W.J., Nezafat R. (2024).

