# Supplementary material for: Myocardial T2 mapping using wideband T2 preparation gradient echo readout for patients with implantable cardiac devices at 1.5T
Source: J Cardiovasc Magn Reson. 2026 Mar 18;28(1):102717. doi: 10.1016/j.jocmr.2026.102717 (PMC13237538; doi:10.1016/j.jocmr.2026.102717)
Supplement: Supplementary file 1 — Supplementary material [file mmc1.docx]

**Supplementary material**


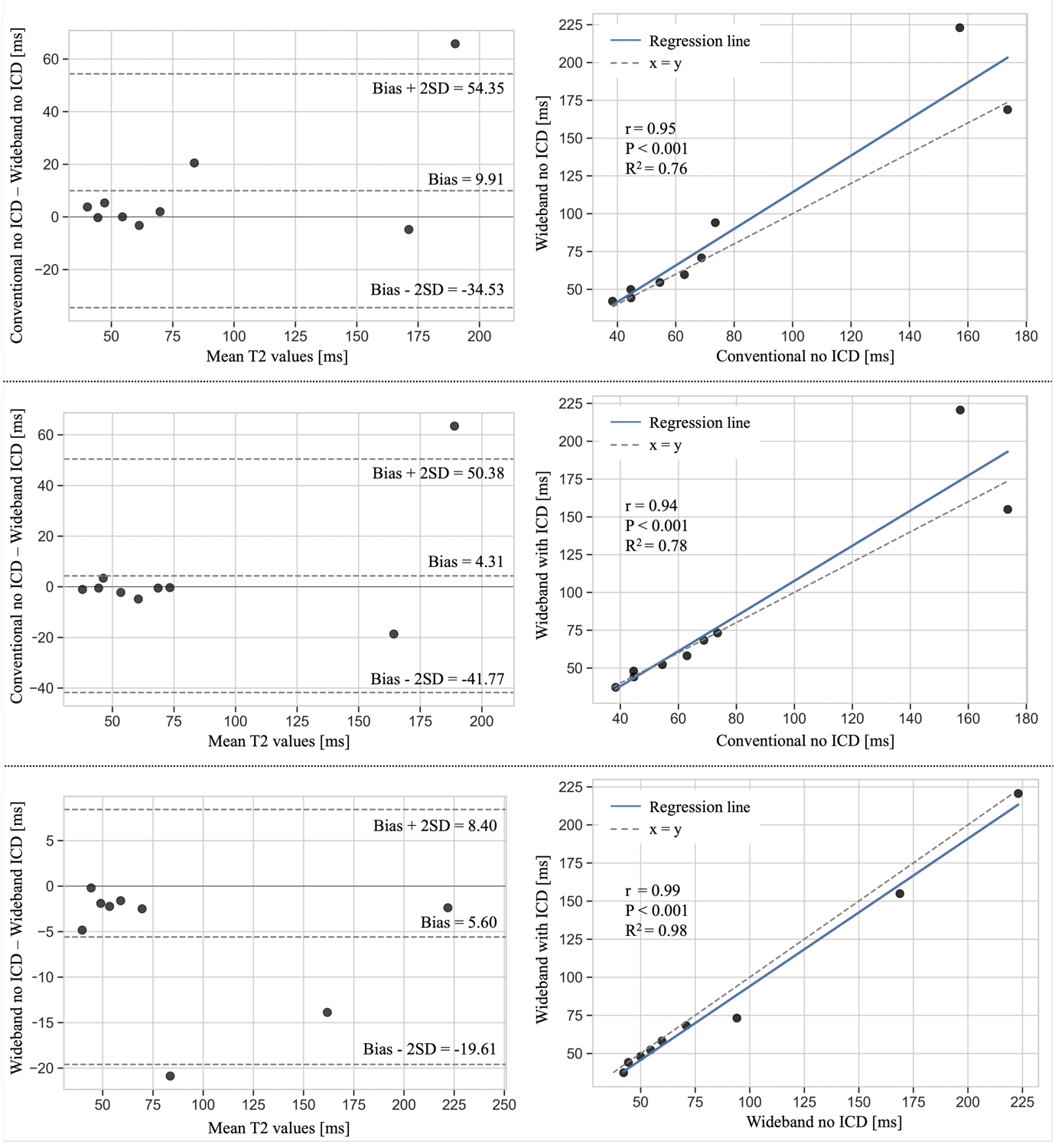


**Supplementary Figure S1**: Results for the phantom study. Left: Bland-Altman analysis comparing the conventional and wideband gradient recalled echo (GRE) mean T2 values, with and without an implantable cardioverter defibrillator (ICD). Right: Pearson’s correlation analysis with correlation coefficients (r) , P-value of the Pearson’s test, and coefficients of determination (R^2^). Abbreviations: SD, standard deviation.

**
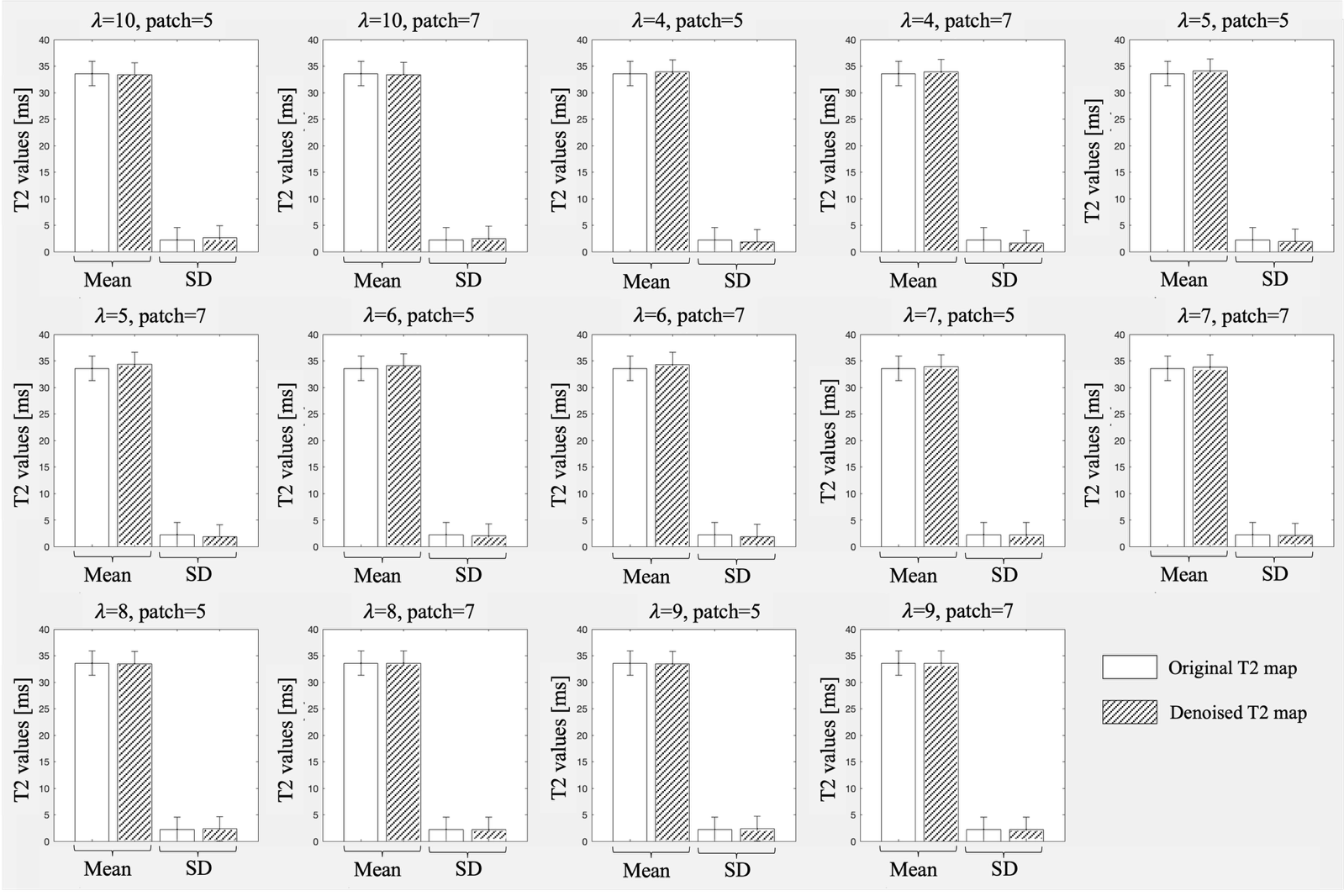
Supplementary Figure S2**: Result in the patient study 1: HD-PROST parameters optimization without ICD. Impact of HD-PROST denoising parameters on the accuracy (mean T2 values) and precision (standard deviations [SD]) of myocardial T2 values in the eight healthy volunteers. Each combination of patch size (5 and 7) and regularization term ($\lambda$=4 to 10) was compared to the non-denoised values (Original T2 map).


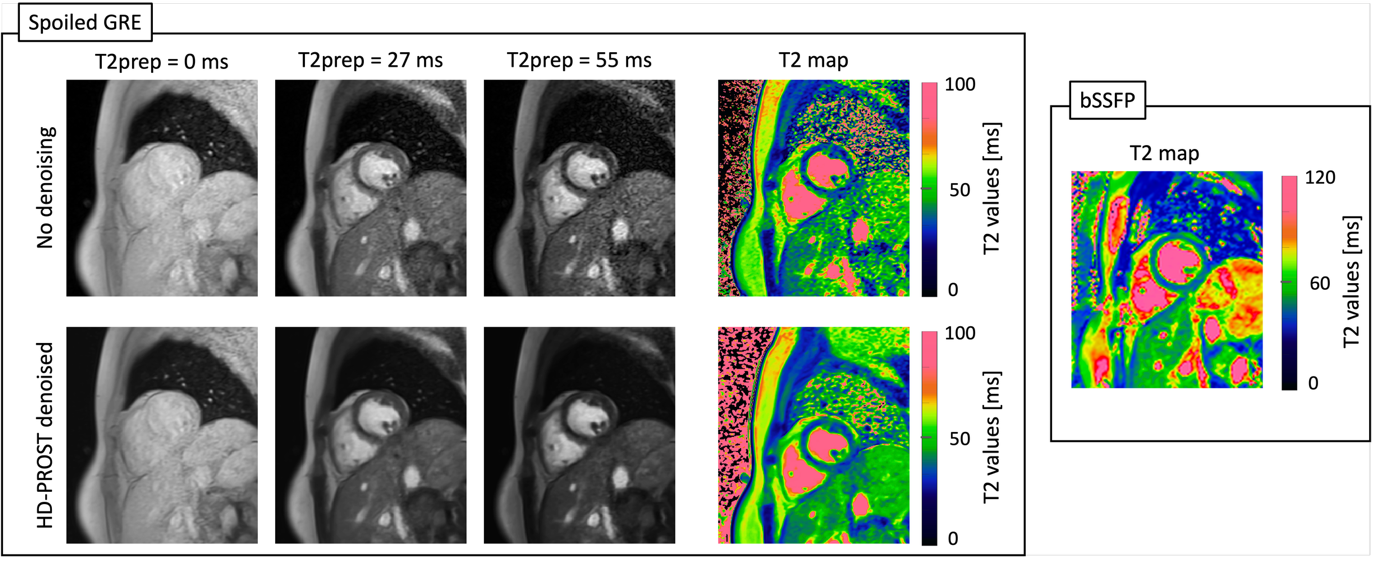


**Supplementary Figure S3**: An example before and after denoising spoiled gradient recalled echo (GRE) T2-weighted images using a regularization term of 4 and a patch size of 7 and resulting calculated T2 maps in a patient with myocardial infarction with non-obstructive coronary arteries. The reference balanced steady-state free-precession (bSSFP) T2 map is shown on the right.


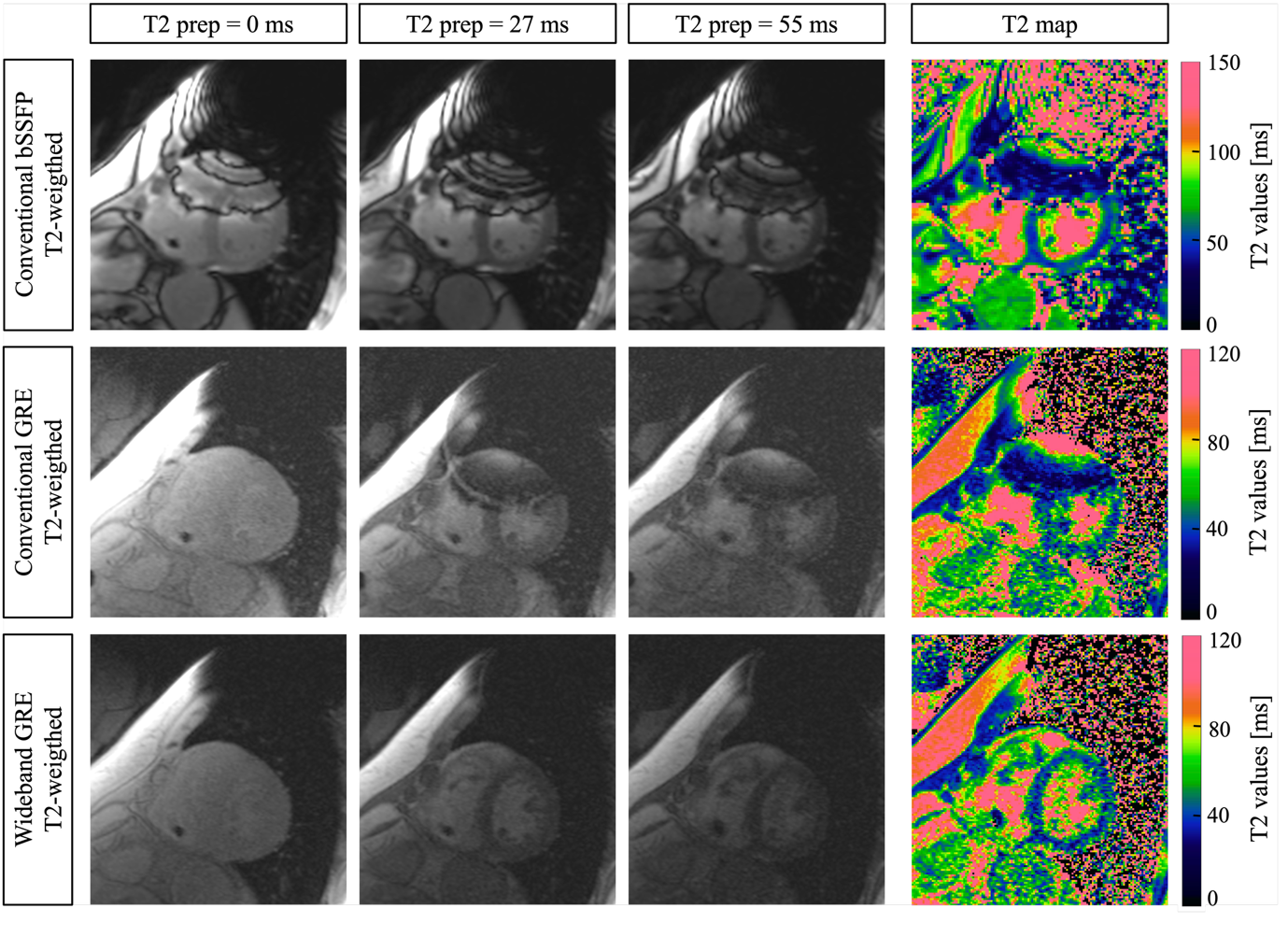


**Supplementary Figure S4**: An example of T2-weigthed images obtained in a patient with a transvenous implantable cardioverter defibrillator (TV-ICD) for the different T2 preparation times (T2 prep) and for conventional balanced steady-state free-precession (bSSFP), conventional spoiled gradient recalled echo (GRE), and wideband GRE. The corresponding calculated T2 maps are shown on the right side.

**Supplementary Table T1**: Result in the patient study 1: HD-PROST parameters optimization without ICD. Results of HD-PROST for each combination of patch size of 5 and 7 and regularization term ($\lambda$) of 4 to 10 on remote mean T2 values and remote T2 standard deviations (SD) compared to the non-denoised values (Original T2 map). A significant P-value is indicated with (*).

| HD-PROST parameters | P-values compared to the non-denoised values | |
| --- | --- | --- |
|  | Remote mean T2 values | Remote SD T2 values |
| $\lambda$ = 4, patch size = 5 | 0.999 | 0.245 |
| $\lambda$ = 4, patch size = 7 | 0.999 | 0.006 (*) |
| $\lambda$ = 5, patch size = 5 | 0.999 | 0.798 |
| $\lambda$ = 5, patch size = 7 | 0.003 (*) | 0.012 (*) |
| $\lambda$ = 6, patch size = 5 | 0.999 | 0.999 |
| $\lambda$ = 6, patch size = 7 | 0.002 (*) | 0.013 (*) |
| $\lambda$ = 7, patch size = 5 | 0.999 | 0.999 |
| $\lambda$ = 7, patch size = 7 | 0.999 | 0.799 |
| $\lambda$ = 8, patch size = 5 | 0.999 | 0.999 |
| $\lambda$ = 8, patch size = 7 | 0.250 | 0.021 (*) |
| $\lambda$ = 9, patch size = 5 | 0.999 | 0.357 |
| $\lambda$ = 9, patch size = 7 | 0.999 | 0.211 |
| $\lambda$ = 10, patch size = 5 | 0.054 | <0.001 (*) |
| $\lambda$ = 10, patch size = 7 | 0.017 (*) | <0.001 (*) |
